# Tumor microenvironment and radioresistance

**DOI:** 10.1038/s12276-021-00640-9

**Published:** 2021-06-16

**Authors:** Tatsuya Suwa, Minoru Kobayashi, Jin-Min Nam, Hiroshi Harada

**Affiliations:** 1 Laboratory of Cancer Cell Biology, Graduate School of Biostudies, Kyoto University, Yoshida Konoe-cho, Sakyo-ku, Kyoto, 606-8501 Japan; 2 Department of Genome Repair Dynamics, Radiation Biology Center, Graduate School of Biostudies, Kyoto University, Yoshida Konoe-cho, Sakyo-ku, Kyoto, 606-8501 Japan; 3 Department of Radiation Oncology and Image-applied Therapy, Graduate School of Medicine, Kyoto University, 54 Shogoin Kawahara-cho, Sakyo-ku, Kyoto, 606-8507 Japan

**Keywords:** Cancer microenvironment, Cancer microenvironment

## Abstract

Metastasis is not the result of a random event, as cancer cells can sustain and proliferate actively only in a suitable tissue microenvironment and then form metastases. Since Dr. Stephen Paget in the United Kingdom proposed the seed and soil hypothesis of cancer metastasis based on the analogy that plant seeds germinate and grow only in appropriate soil, considerable attention has focused on both extracellular environmental factors that affect the growth of cancer cells and the tissue structure that influences the microenvironment. Malignant tumor tissues consist of not only cancer cells but also a wide variety of other cells responsible for the inflammatory response, formation of blood vessels, immune response, and support of the tumor tissue architecture, forming a complex cellular society. It is also known that the amounts of oxygen and nutrients supplied to each cell differ depending on the distance from tumor blood vessels in tumor tissue. Here, we provide an overview of the tumor microenvironment and characteristics of tumor tissues, both of which affect the malignant phenotypes and radioresistance of cancer cells, focusing on the following keywords: diversity of oxygen and nutrient microenvironment in tumor tissue, inflammation, immunity, and tumor vasculature.

## Cancer: Exploring tumor microenvironments and radioresistance

Extensive research into diverse tumor microenvironments in all cancer types is needed to inform novel therapeutic strategies. For cancer to metastasize, the conditions of the surrounding tumor microenvironment must be just right; this ‘seed and soil’ hypothesis has prompted recent research into extracellular factors influencing cancer behavior. Hiroshi Harada at Kyoto University in Japan and co-workers reviewed current understanding of tumor microenvironments and their influence on radiation resistance. Every tumor displays a unique microenvironment comprising multiple cell types involved in complex crosstalk, and the composition of these cellular communities influences inflammation, blood vessel formation and immune responses. On acute and chronic timescales, the levels of oxygen and nutrients reaching cancer cells varies widely across tumor regions, dynamically influencing character of cancer cells. The presence of diverse cellular society and oxygen-poor tumor regions eventually appear to reduce radiotherapy success.

## Heterogeneous oxygen microenvironment in malignant solid tumors and radioresistance of cancer cells

### Tumor hypoxia

Tumor hypoxia has attracted marked attention in radiation biology and oncology since Thomlinson and Gray reported the presence of hypoxic cells in malignant solid tumors and suggested the possibility that they exert a negative impact on the outcome of radiation therapy.

**Fig. 1:**
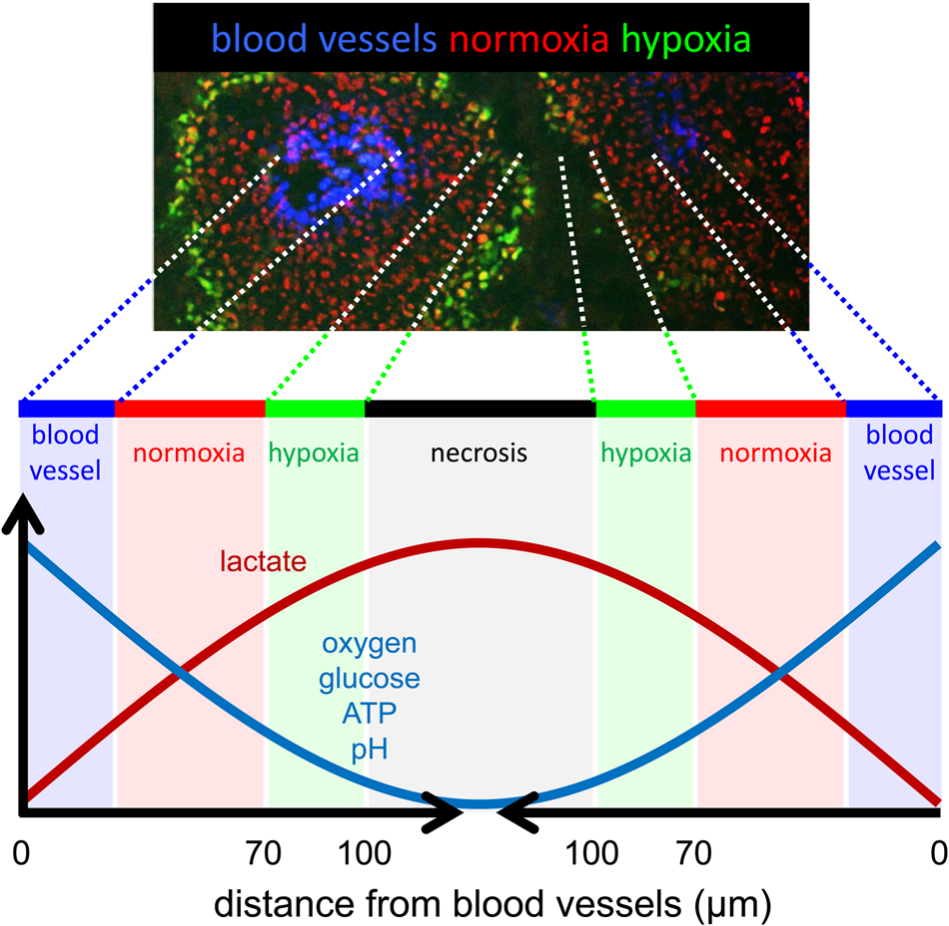
Schematic diagram of microenvironments in and among tumor cords. Because of the limited distance that molecular oxygen diffuses from functional tumor blood vessels, pO_2_ gradually decreases according to the distance from the vessel, leading to the generation of chronically hypoxic and necrotic regions. The availability of nutrients, such as glucose, and the pH are relatively low in these regions.

Activation of oncogenes and inactivation of tumor suppressor genes cause aberrant cell proliferation, which is a characteristic feature of cancer cells. Aberrant cell proliferation leads to an oxygen supply–demand imbalance in cancer cells and an imbalance between the rate of vascular network development and that of cancer cell proliferation in tumor tissues^1,2^. These are recognized as two major causative factors of the heterogeneous cellular oxygenation in tumor tissues (Fig. 1). It is reasonable to consider that cancer cells can obtain sufficient oxygen and nutrients only when in close proximity to tumor blood vessels (normoxic regions), because molecular oxygen can diffuse within a limited distance from functional blood vessels (Fig. 1). On the other hand, cancer cells approximately 70–100 μm from tumor blood vessels can obtain the minimal level of oxygen necessary for their survival but insufficient for active proliferation (chronically hypoxic regions; Fig. 1). Yeom et al. previously suggested that hypoxic layers approximately 70–85 μm from tumor blood vessels are relatively mildly hypoxic, with O_2_ concentrations of at least 1% but no higher than 3%^3^. On the other hand, the concentration of O_2_ approximately 85–100 μm from tumor blood vessels has been suggested to be 1% or less^3^. Cancer cells in hypoxic regions are known to produce adenosine triphosphate (ATP) mainly via oxygen-independent glycolysis rather than oxygen-dependent oxidative phosphorylation and generate lactate as a byproduct, leading to a unique microenvironment with a low pH. Cancer cells farther away from blood vessels inevitably die because they are deprived of oxygen and nutrients (necrotic regions; Fig. 1)^2–4^. A dynamic oxygen concentration gradient also exists in normal tissues, such as between the portal and central veins in liver tissue; however, a necrotic region is a unique and characteristic feature of malignant solid tumors (Fig. 1).

In addition to chronic hypoxia, another kind of hypoxia is known to exist in tumor tissues: so-called acute hypoxia. Acute hypoxia is generated—regardless of the distance from tumor blood vessels—as a result of unusual activation of proangiogenic mechanisms mediated by a hypoxia-responsive transcription factor, hypoxia-inducible factor 1 (HIF-1). Cancer cells adapt to hypoxic conditions by inducing the expression of HIF-1. HIF-1 induces the expression of various proangiogenic factors, such as vascular endothelial growth factor (VEGF) and platelet-derived growth factor (PDGF). Activation of HIF-1 in tumor hypoxia causes excess activation of proangiogenic signals, resulting in the development of unique vasculatures that are structurally tortuous, immature, and functionally very leaky due to poor coverage by pericytes. Interstitial pressure is inevitably high in solid tumors because of this leakiness; therefore, tumor blood vessels cannot sufficiently supply oxygen and nutrients even in well-vascularized regions. In addition, these immature tumor vessels cause transient reflux and occlusion of blood flow, also leading to transient hypoxia even within 70 μm of tumor blood vessels (acutely hypoxic regions)^2,3^. The concept of acute hypoxia was first recognized by Brown et al. in 1979^5^. Subsequent studies showed that at least 20% of cancer cells are subjected to acute hypoxia^6–8^.

It has been reported in clinical studies that the overall median pO_2_ measured by a computerized polarographic needle electrode is approximately 10 mmHg in various malignant solid tumors, such as breast cancers and head and neck cancers^9^. In contrast, that in normal tissues has been reported to be approximately 65 mmHg. It has also been reported that the overall hypoxic fraction (pO_2_ < 2.5 mmHg) is approximately 25% in breast cancer, while pO_2_ values lower than 12.5 mmHg have never been noted in normal breast tissues^10^.

### Mechanisms underlying tumor radioresistance under hypoxia

Through basic experiments, such as the clonogenic cell survival assay, it has been repeatedly confirmed that cultured cells are approximately 2–3 times more radiosensitive under normoxia than under hypoxia^11^. Consistent with this phenomenon, known as the “oxygen effect” in the field of radiation biology, accumulated clinical evidence has also demonstrated that a large hypoxic fraction in a solid tumor is correlated with poor prognosis in cancer patients after radiation therapy^11,12^. For example, Kaanders et al. reported that locoregional tumor control was significantly lower for patients with head and neck cancers containing more hypoxic regions^12^. The cytotoxic effects of radiation are principally caused by damage to genomic DNA produced through either direct ionization of DNA or indirect action by creating water radical species that react with DNA. The oxygen effect is known to mainly result from the difference in the frequency of DNA damage produced through indirect action under hypoxia compared with normoxia.

#### Radiochemical mechanism

Ionizing radiation, such as X-rays and γ-rays, produces free radicals through ionization of water. When highly reactive radicals such as hydroxyl radicals (HO•) are produced from water near genomic DNA, they cause DNA strand breaks. Molecular oxygen can stabilize reactive radicals, enabling them to cause severe DNA damage for a long period of time. In addition, molecular oxygen oxidizes the ends of damaged DNA and creates double-strand breaks that are both unrepairable and lethal^11,13^. Therefore, DNA damage is less severe in the absence of oxygen^14^, and cancer cells in hypoxic regions exhibit radioresistance^2^.

#### Radiobiological mechanism

Biological as well as chemical mechanisms are important elements contributing to the radioresistance of hypoxic cancer cells^15–17^. One of the important mediators of these mechanisms is HIF-1. In particular, the function of HIF-1 in carbohydrate metabolic reprogramming from mitochondrial oxidative phosphorylation to accelerated glycolysis and the associated pentose phosphate pathway (PPP) is important for the radioresistance of hypoxic cancer cells^2,18,19^. The PPP, whose initial metabolite, glucose-6-phosphate, is supplied via glycolysis, generates pentose sugars and ribose-5-phosphate and, moreover, generates nicotinamide adenine dinucleotide phosphate (NADPH) as a byproduct^20,21^. Because NADPH is important for the production of an antioxidant, reduced glutathione (GSH), from glutathione-S-S-glutathione (GSSG) and because ribose-5-phosphate is important for nucleotide synthesis, HIF-1-mediated activation of the PPP is associated with the radioresistance of hypoxic cancer cells^22^.

**Fig. 2:**
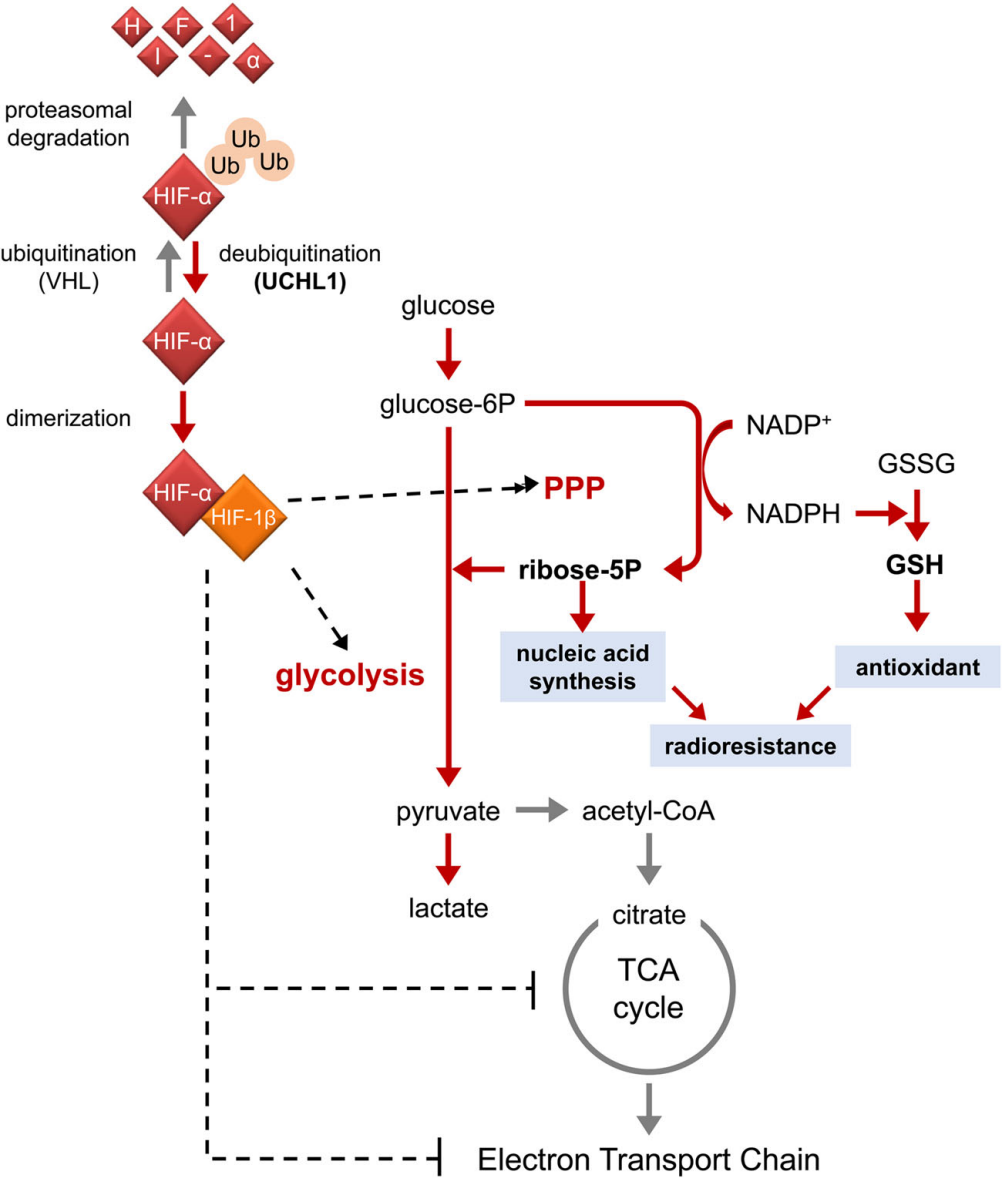
Radioresistance of cells induced by UCHL1-HIF-1 pathway-dependent reprogramming of the carbohydrate metabolic pathway. HIF-1 mediates reprogramming of the glucose metabolic pathway from mitochondrial oxidative phosphorylation to glycolysis. UCHL1 causes radioresistance by inducing HIF-1-mediated reprogramming and a resulting increase in the intracellular level of GSH in a PPP-dependent manner.

We recently demonstrated the possibility that aberrant activation of upstream activators of HIF-1, such as a deubiquitinating enzyme for HIF-1α protein, ubiquitin carboxyl-terminal hydrolase-L1 (UCHL1), induces the same HIF-1-dependent mechanism for radioresistance even under normoxic conditions (Fig. 2). For example, UCHL1 was found to increase the intracellular levels of GSH and NADPH even under normoxic conditions through HIF-1-mediated reprogramming of carbohydrate metabolism, specifically in a PPP-dependent manner. As a result, UCHL1 induced radioresistance even under normoxic conditions through UCHL1-HIF-1-mediated metabolic reprogramming^19,23^. Consistent with this finding, accumulated clinical evidence has shown that overexpression of the HIF-1 protein in a solid tumor is associated with a poor prognosis in cancer patients with, e.g., head and neck, cervical, or oropharyngeal cancer after irradiation^24–26^.

Extensive studies have shown that radioresistance of hypoxic cancer cells is induced by the ataxia-telangiectasia mutated (ATM)-dependent DNA damage repair pathway, particularly through upregulation of homologous recombination activity^27–32^. In response to DNA strand breaks, the activated form of ATM phosphorylates some HR-related factors, such as breast cancer type 1 susceptibility gene (BRCA1) and Fanconi anemia group D2 (FANCD2), triggering the DNA damage repair pathway. Because ATM expression is known to be induced also in response to hypoxic stress, it has been suggested that ATM also plays pivotal roles in the radioresistance of cancer cells under hypoxic conditions.

Another mechanism underlying the radioresistance of hypoxic cells is related to immune evasion. Because radiation therapy damages cancer cells and potentiates the production of cancer neoantigens, the therapeutic effect of radiation is greatly influenced by tumor immunity. Cancer cells express immune inhibitory molecules, such as PD-L1, and escape tumor immunity. PD-L1 expression is induced under hypoxic conditions in a HIF-1-dependent manner and is thus thought to be one of the important mechanisms underlying the radioresistance of hypoxic cancer cells^33^. Thus, immune checkpoint inhibitors are expected to enhance the therapeutic effect of radiation on tumor tissues containing more hypoxic regions. Immune checkpoint inhibitors have been reported to induce vascular normalization and thus decrease the hypoxic fraction^34,35^; this is another reason that we expect immune checkpoint inhibitors to enhance the therapeutic effect of radiation.

## Cellular society within a malignant solid tumor

**Fig. 3:**
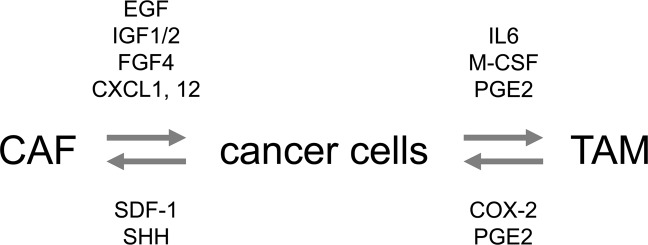
Crosstalk between cancer cells and TAMs or CAFs in tumor tissue. Crosstalk between cancer cells and TAMs or CAFs is regulated by the indicated factors.

An inflammatory reaction is constantly evoked in most malignant solid tumors, which causes infiltration of immune cells, angiogenesis, and proliferation of fibroblasts and produces a characteristic cellular society (Fig. 3). It has been clarified that the formation of this cellular society leads to the malignant progression and radioresistance of cancers^36,37^.

### Radioresistance of cancers mediated by tumor-associated macrophages (TAMs)

Tumor-associated macrophages (TAMs), which exist abundantly in tumor tissues, have long been considered to exhibit antitumor effects and suppress tumor growth. However, recent studies have shown that they promote tumor growth and enhance radioresistance in various types of cancers, including breast, esophageal, cervical, and hepatic cancers^37–39^. Macrophages induce the expression of the inflammatory prostaglandin synthase cyclooxygenase-2 (COX-2) via the activation of nuclear factor κB (NF-κB), and the produced prostaglandin E2 (PGE2) has been reported to then accelerate tumor growth and induce radioresistance in cancers^40,41^. In addition, it has been suggested that macrophages activated by irradiation may cause radioresistance of cancer cells and facilitate tumor recurrence after radiotherapy by inducing high expression of tumor necrosis factor-α (TNF-α) and promoting angiogenesis^42^.

The crosstalk between cancer cells and macrophages plays an important role in the growth and radioresistance of tumors. Macrophages differentiate into the M2 type when stimulated with interleukin 6 (IL6), macrophage colony stimulating factor (M-CSF), and PGE2 secreted by cancer cells. Next, proangiogenic factors, e.g., VEGF and PDGF, secreted from M2-type macrophages have been suggested to generate a microenvironment favorable for tumor growth^43^ and promote tumor regrowth after radiotherapy. It has also been pointed out that antitumor immunity is suppressed by the infiltration of regulatory T cells induced by C–C motif chemokine ligand 22 (CCL22) secreted from M2-type macrophages^39,44^, which leads to increased tumor growth and cancer radioresistance^45–48^.

### Radioresistance of cancers mediated by cancer-associated fibroblasts (CAFs)

Clinical studies on various types of cancers, such as head and neck, lung, pancreatic, and rectal cancers, have reported that the prognosis of patients after radiotherapy is poorer when tumor tissues contain more fibroblasts^49–56^. This clinical evidence led to two major questions: how such a fibroblast-rich microenvironment is created in tumor tissues, and how it enhances radioresistance. Some important findings have been reported that suggest the importance of crosstalk between cancer cells and fibroblasts in tumor tissues.

PDGF and transforming growth factor-β (TGF-β), which are secreted from cancer cells, are known to induce transdifferentiation of stromal fibroblasts into myofibroblasts, called “cancer-associated fibroblasts (CAFs)”, when stromal fibroblasts preexist in or are recruited into tumor tissue^57–59^. Moreover, TNF-α produced by macrophages has been reported to stimulate the proliferation of fibroblasts in the process of wound healing and acts similarly in tumor tissue to create a fibroblast-rich tumor microenvironment^60–62^. Since the interior region of a tumor rich in fibroblasts is known to contain an abundance of hypoxic regions, a CAF-rich microenvironment is considered to cause radioresistance.

Recent studies, including ours, have revealed the existence of a positive feedback loop by which both CAFs and hypoxic tumor cells enhance the generation of each other^63,64^ and have indicated the importance of this feedback loop in tumor radioresistance. We reported increased expression and secretion of Sonic hedgehog (SHH) protein in pancreatic cancer cells upon exposure to hypoxic stimuli. This increased transcription of SHH mRNA and secretion of SHH protein were observed to be HIF-1 dependent. SHH protein secreted from pancreatic cancer cells under hypoxia indeed stimulated the Sonic hedgehog signaling pathway in fibroblasts and promoted their growth in a paracrine manner^63^. Such a positive feedback loop may account for the characteristic of pancreatic cancer as the most aggressive malignancy; it is rich in both fibroblasts and hypoxic regions compared with other types of cancer tissues^65^ and is therefore resistant to radiotherapy^66^.

In addition, it has been reported that some factors secreted from CAFs induce radioresistance in cancer cells. For example, CAFs secrete epidermal growth factor (EGF), insulin-like growth factor 2 (IGF2), and fibroblast growth factor 4 (FGF4), which increase not only the proliferation but also the survival of cancer cells after irradiation^67^. It has also been reported that IGF1/2, C-X-C motif chemokine ligand 12 (CXCL12; also called stromal cell-derived factor 1, SDF-1), and β-hydroxybutyrate secreted from CAFs induce autophagy in irradiated cancer cells and accelerate the recovery and regrowth of tumors after irradiation^68^. In addition, CXCL1 secreted from CAFs was found to cause the accumulation of reactive oxygen species (ROS) in irradiated cancer cells by inhibiting the ROS-scavenging enzyme superoxide dismutase 1 (SOD1), leading to enhanced DNA damage repair in cancer cells^69^. These are just a few examples, and there are other mechanisms responsible for the promotive effects of CAFs on tumor radioresistance.

## Discussion

As summarized here, tumor microenvironments are extremely diverse and significantly different from the microenvironments of normal tissues. For example, not only cancer cells but also a wide variety of other cells are present within malignant solid tumors, forming a unique cellular society. In addition, the oxygen and nutrient environments are not uniform in tumor tissue due to the tumor vascular architecture. Furthermore, there are differences in the tumor microenvironment among cancer types; e.g., kidney cancers are hypervascular^70,71^, and pancreatic cancers are fibrotic/fibroblast-rich^66^. It has become clear in recent years that these environmental factors interact with each other and create a complex intratumoral microenvironment. For the development of novel therapeutic strategies for cancers, it is necessary to understand the nature of the seed and soil hypothesis by identifying the characteristics of tumor microenvironments in a spatiotemporal and global manner and by elucidating the mechanism underlying the induction of malignant phenotypes and therapeutic resistance in cancers.
